# Conferência sobre Mudança do Clima COP27: ações urgentes e necessárias para a África e o mundo^*^

**DOI:** 10.26633/RPSP.2022.220

**Published:** 2022-11-18

**Authors:** Lukoye Atwoli, Gregory E. Erhabor, Aiah A. Gbakima, Abraham Haileamlak, Jean-Marie Kayembe Ntumba, James Kigera, Laurie Laybourn-Langton, Bob Mash, Joy Muhia, Fhumulani Mavis Mulaudzi, David Ofori-Adjei, Friday Okonofua, Arash Rashidian, Maha El-Adawy, Siaka Sidibé, Abdelmadjid Snouber, James Tumwine, Mohammad Sahar Yassien, Paul Yonga, Lilia Zakhama, Chris Zielinski

**Affiliations:** 1 Editor-chefe, East African Medical Journal Editor-chefe, East African Medical Journal; 2 Editor-chefe, West African Journal of Medicine Editor-chefe, West African Journal of Medicine; 3 Editor-chefe, Sierra Leone Journal of Biomedical Research Editor-chefe, Sierra Leone Journal of Biomedical Research; 4 Editor-chefe, Ethiopian Journal of Health Sciences Editor-chefe, Ethiopian Journal of Health Sciences; 5 Editor chefe, Annales Africaines de Médecine Editor chefe, Annales Africaines de Médecine; 6 Editor-chefe, Annals of African Surgery Editor-chefe, Annals of African Surgery; 7 Universidade de Exeter Universidade de Exeter; 8 Editor-chefe, African Journal of Primary Health Care & Family Medicine Editor-chefe, African Journal of Primary Health Care & Family Medicine; 9 Escola de Higiene e Medicina Tropical de Londres Escola de Higiene e Medicina Tropical de Londres; 10 Editora-chefe, Curationis Editora-chefe, Curationis; 11 Editor-chefe, Ghana Medical Journal Editor-chefe, Ghana Medical Journal; 12 Editor-chefe, African Journal of Reproductive Health Editor-chefe, African Journal of Reproductive Health; 13 Editor Executivo, Eastern Mediterranean Health Journal Editor Executivo, Eastern Mediterranean Health Journal; 14 Diretora de Promoção da Saúde Eastern Mediterranean Health Journal Diretora de Promoção da Saúde, Eastern Mediterranean Health Journal; 15 Diretor de Publicação Mali Médical Diretor de Publicação, Mali Médical; 16 Editor-chefe, Journal de la Faculté de Médecine d'Oran Editor-chefe, Journal de la Faculté de Médecine d'Oran; 17 Editor-chefe, African Health Sciences Editor-chefe, African Health Sciences; 18 Editor-chefe, Evidence-Based Nursing Research Editor-chefe, Evidence-Based Nursing Research; 19 Editor-chefe, East African Medical Journal Editor-chefe, East African Medical Journal; 20 Editora-chefe, La Tunisie Médicale Editora-chefe, La Tunisie Médicale; 21 Universidade de Winchester Universidade de Winchester

## AS NAÇÕES RICAS DEVEM AUMENTAR O APOIO À ÁFRICA E AOS PAÍSES VULNERÁVEIS PARA ENFRENTAR OS IMPACTOS PASSADOS, PRESENTES E FUTUROS DA MUDANÇA DO CLIMA.

O relatório de 2022 do Painel Intergovernamental sobre Mudança do Clima (IPCC) pinta um quadro sombrio para o futuro da vida na Terra, caracterizado pelo colapso dos ecossistemas, extinção de espécies e riscos climáticos, tais como ondas de calor e inundações (1). Todos eles estão ligados a problemas de saúde física e mental, com consequências diretas e indiretas no aumento da morbidade e da mortalidade. Para evitar esses efeitos catastróficos na saúde em todas as regiões do mundo, há um amplo consenso – como argumentaram juntos 231 periódicos de saúde em 2021 – de que o aumento da temperatura global deve ser limitado a menos de 1,5 ^o^C em comparação com os níveis pré-industriais.

Embora o Acordo de Paris de 2015 delineie uma estrutura de ação global que incorpora o financiamento climático aos países em desenvolvimento, esse apoio ainda não se concretizou (2). A COP27 é a quinta Conferência das Partes (COP) a ser organizada na África desde seu início, em 1995. Antes dessa reunião, nós, editores de periódicos de saúde de todo o continente, pedimos uma ação urgente para garantir que esta seja a COP que finalmente proporcione justiça climática para a África e os países vulneráveis. É algo essencial não apenas para a saúde desses países, mas para a saúde de todo o mundo.

## A ÁFRICA TEM SOFRIDO DESPROPORCIONALMENTE, EMBORA POUCO TENHA CONTRIBUÍDO PARA CAUSAR A CRISE

A crise climática teve um impacto nos determinantes ambientais e sociais da saúde em toda a África, levando a efeitos devastadores na saúde (3). Esses impactos podem ser resultado direto de choques ambientais e indireto de efeitos mediados socialmente (4). Os riscos relacionados à mudança do clima na África incluem inundações, secas, ondas de calor, redução da produção de alimentos e redução da produtividade laboral (5).

As secas na África subsaariana triplicaram entre 1970-1979 e 2010-2019 (6). Em 2018, ciclones devastadores atingiram três milhões de pessoas no Malawi, em Moçambique e em Zimbábue (6). Na África ocidental e central, inundações severas resultaram em mortalidade e migração forçada por perda de abrigo, terras cultivadas e gado (7). As mudanças na ecologia de vetores provocadas pelas enchentes e danos à higiene ambiental levaram ao aumento de doenças na África subsaariana, com aumento de malária, dengue, febre de Lassa, febre do Vale do Rift, doença de Lyme, vírus Ebola, vírus do Nilo Ocidental e outras infecções (8, 9). A elevação do nível do mar reduz a qualidade da água, levando a doenças transmitidas pela água, incluindo doenças diarreicas – uma das principais causas de mortalidade na África (8). O clima extremo prejudica o abastecimento de água e alimentos, aumentando a insegurança alimentar e a desnutrição, o que causa 1,7 milhão de mortes anuais na África (10). De acordo com a Organização das Nações Unidas para a Alimentação e a Agricultura, a desnutrição aumentou quase 50% desde 2012, devido ao papel central que a agricultura desempenha nas economias africanas (11). Os choques ambientais e seus efeitos colaterais também causam sérios danos à saúde mental (12). No total, estima-se que a crise climática destruiu um quinto do produto interno bruto (PIB) dos países mais vulneráveis aos choques climáticos (13).

Os danos à África devem ser de suprema preocupação para todas as nações, em parte por razões morais. É altamente injusto que as nações mais impactadas tenham contribuído menos para as emissões cumulativas globais que estão provocando a crise climática e seus efeitos cada vez mais severos. A América do Norte e a Europa contribuíram com 62% das emissões de dióxido de carbono desde a Revolução Industrial, enquanto a África contribuiu com apenas 3% (14).

## A LUTA CONTRA A CRISE CLIMÁTICA PRECISA DO EMPENHO DE TODOS

Não é apenas por razões morais que todas as nações devem se preocupar com a África. Os impactos agudos e crônicos da crise climática criam problemas como pobreza, doenças infecciosas, migração forçada e conflitos que se espalham pelos sistemas globalizados (6, 15). Esses impactos com efeito cascata afetam todas as nações. A COVID-19 serviu como um alerta para essas dinâmicas globais, e não é uma coincidência que os profissionais de saúde tenham sido ativos na identificação e resposta às consequências dos crescentes riscos sistêmicos para a saúde. Mas as lições da pandemia de COVID-19 não devem ser limitadas ao risco pandêmico (16, 17). É imperativo que o sofrimento das nações na linha de frente, incluindo as da África, seja a consideração central na COP27: em um mundo interligado, deixar os países à mercê dos choques ambientais cria instabilidade com consequências severas para todas as nações.

O foco principal das cúpulas climáticas continua sendo a redução rápida das emissões para que os aumentos da temperatura global sejam mantidos abaixo de 1,5 °C, o que limitará os danos. Mas, para a África e outras regiões vulneráveis, esse dano já é grave. Atingir a meta prometida de fornecer 100 bilhões de dólares de financiamento climático por ano é agora globalmente crítico se quisermos evitar os riscos sistêmicos de deixar as sociedades em crise. Pode-se alcançar isso ao garantir que esses recursos se concentrem em aumentar a resiliência aos impactos já existentes e futuros que são inevitáveis da crise climática, bem como ao apoiar nações vulneráveis na redução das emissões de gases de efeito estufa: uma paridade de estima entre adaptação e mitigação. Esses recursos devem ser oriundos de subsídios e não de empréstimos, e ser urgentemente aumentados antes do atual período de revisão de 2025. Eles devem colocar a resiliência do sistema de saúde em primeiro plano, uma vez que as crises agravantes causadas pela crise climática muitas vezes se manifestam em problemas de saúde agudos. Financiar a adaptação será mais econômico do que depender de medidas de alívio de desastres.

Alguns progressos foram alcançados na adaptação na África e no mundo todo, incluindo sistemas de alerta precoce e infraestrutura de defesa contra eventos extremos. Mas as nações na linha de frente não são compensadas pelos impactos de uma crise que não causaram. Não só é injusto, mas também impulsiona a espiral de desestabilização global, uma vez que as nações gastam dinheiro para responder a desastres mas não conseguem mais pagar por uma maior resiliência ou para reduzir o problema de base por meio da redução de emissões. Um mecanismo de financiamento para perdas e danos deve ser agora introduzido, fornecendo recursos adicionais além daqueles concedidos para mitigação e adaptação. Deve-se ir além das falhas da COP26, em que a sugestão de tal mecanismo foi rebaixada para "um diálogo" (18).

A crise climática é um produto da inação global e tem um grande custo não só para os países africanos impactados desproporcionalmente, mas para o mundo inteiro.. A África se junta a outras regiões na linha de frente para exortar as nações ricas a finalmente darem um passo adiante. Caso contrário, mais cedo ou mais tarde as crises na África se espalharão e engolirão todas as regiões do mundo, e então pode ser tarde demais para responder efetivamente. Se até hoje não foi possível persuadi-las com argumentos morais, espera-se que agora seus interesses próprios prevaleçam.
